# Effects of blue-light LED toothbrush on reducing dental plaque and gingival inflammation in orthodontic patients with fixed appliances: a crossover randomized controlled trial

**DOI:** 10.1186/s12903-023-02977-1

**Published:** 2023-05-15

**Authors:** Chavirakarn Manphibool, Oranart Matangkasombut, Soranun Chantarangsu, Pintu-on Chantarawaratit

**Affiliations:** 1 Department of Orthodontics, Faculty of Dentistry, Chulalongkorn University, Bangkok, Thailand; 2 Department of Microbiology and Center of Excellence on Oral Microbiology and Immunology, Faculty of Dentistry, Chulalongkorn University, Bangkok, Thailand; 3 Department of Oral Pathology, Faculty of Dentistry, Chulalongkorn University, Bangkok, Thailand

**Keywords:** Biofilm, Dental plaque, Fixed orthodontic appliance, Gingival index, Gingival inflammation, LED toothbrush, Orthodontic patient, Plaque index, *Streptococcus mutans*

## Abstract

**Background:**

Patients with fixed orthodontic appliances have higher plaque accumulation and gingival inflammation. Our aim was to compare the effectiveness of a light emitting diode (LED) toothbrush with a manual toothbrush in reducing dental plaque and gingival inflammation in orthodontic patients with fixed appliances, and to investigate the effect of the LED toothbrush on *Streptococcus mutans* (*S. mutans*) biofilm in vitro.

**Methods:**

Twenty-four orthodontic patients were recruited and randomly assigned into 2 groups: (1) started with manual and (2) started with LED toothbrushes. After a 28-day usage and 28-day wash-out period, the patients switched to the other intervention. The plaque and gingival indices were determined at baseline and 28 days after each intervention. The patients’ compliance and satisfaction scores were collected using questionnaires. For the in vitro experiments, *S. mutans* biofilm was divided into 5 groups (n = 6) with 15-, 30-, 60-, or 120-sec LED exposure, and without LED exposure as a control group.

**Results:**

There was no significant difference in the gingival index between the manual and LED toothbrush groups. The manual toothbrush was significantly more effective in reducing the plaque index in the proximal area on the bracket side (*P* = 0.031). However, no significant difference was found between the two groups in other areas around the brackets or on the non-bracket side. After LED exposure in vitro, the percentages of bacterial viability after LED exposure for 15–120 s were significantly lower compared with the control (*P* = 0.006).

**Conclusion:**

Clinically, the LED toothbrush was not more effective in reducing dental plaque or gingival inflammation than the manual toothbrush in orthodontic patients with fixed appliances. However, the blue light from the LED toothbrush significantly reduced the number of *S. mutans* in biofilm when it was exposed to the light for at least 15 s in vitro.

**Clinical Trial Registration:**

Thai Clinical Trials Registry (TCTR20210510004). Registered 10/05/2021.

**Supplementary Information:**

The online version contains supplementary material available at 10.1186/s12903-023-02977-1.

## Background

Dental plaque, a structurally and functionally organized biofilm with a diverse microbial composition, is one of the main etiological factors of dental caries and periodontal disease [1]. Fixed orthodontic appliances are a complex apparatus composed of brackets, archwires, and other auxiliary devices that are likely to be plaque retentive areas and limit effective oral hygiene home care [2]. Studies have indicated that patients with fixed orthodontic appliances have higher plaque accumulation [3, 4]. Therefore, this dental plaque can lead to enamel demineralization [1–3], gingival inflammation, and bleeding on probing values [5–9]. Furthermore, the plaque index (PI) and gingival index (GI) reached their maximum values after 3 months of fixed appliance placement [9].

At the microbiological level, placing fixed orthodontic appliances cause a microbial shift towards more pathogenic bacteria, such as streptococci and lactobacilli, which are cariogenic [6, 9]. The colonization of periodontal pathogens in the gingival crevices also escalates [7]. Increases in anaerobic pathogenic bacteria, such as *Tannerella forsythia*, *Campylobacter rectus*, and *Prevotella nigrescens*, were found in the leveling and alignment phases of fixed orthodontic treatment [6, 7]. Due to the difficulties in maintaining adequate oral hygiene during treatment, 10% of post-orthodontic patients experienced more clinical attachment loss compared with the no treatment group [10].

Toothbrushes together with complementary aids (e.g. dental floss, single-tufted brushes, and interdental brushes) and mouthwash are highly recommended for domestic plaque removal in orthodontic patients [11–13]. However, due to the lack of brushing skills and adequate patient cooperation, dental plaque control remains a significant challenge for orthodontic patients with fixed appliances. To resolve this issue, innovative technologies have been introduced to promote oral hygiene.

Antimicrobial photodynamic therapy (aPDT) has been used for decades to treat dental caries, endodontic disinfection, oral candidiasis, and periodontal and peri-implant disease [14–21]. In addition, blue light from a light emitting diode (LED) has a bactericidal effect on *Streptococcus mutans* (*S. mutans*) and oral biofilms [15, 22–24]. The lethal effect of blue light on *S. mutans* biofilm was seen after a 7- or 10-min exposure and most of the bacteria that were killed were on the outer surface of the biofilm [22].

An LED toothbrush was introduced that added aPDT using blue light on dental plaque [16, 25–27]. Clinically, the blue LED toothbrush with a 412 nm wavelength significantly reduced dental plaque, gingival bleeding, and inflammation more than the manual toothbrushes [16]. However, research in this area remains limited, and no study has been performed in orthodontic patients with fixed appliances. We hypothesized that the LED toothbrush may be more effective in reducing GI and PI scores. Thus, the aim of this study was to investigate the effectiveness of an LED toothbrush in removing dental plaque and reducing gingival inflammation in orthodontic patients with fixed appliances compared with using a manual toothbrush, and to evaluate the bactericidal effect of the LED toothbrush on *S. mutans* biofilm in vitro.

## Methods

### Objectives

The primary objective of the present study was to compare the effectiveness of an LED toothbrush with a manual toothbrush in orthodontic patients with fixed appliances after 28 days of brushing 2 times/day, as assessed by the GI and PI scores. The secondary objective was to determine the effective duration of LED toothbrush exposure on *Streptococcus mutans* (*S. mutans*) biofilm in vitro. The exploratory objectives were to investigate the influence of patients’ age, satisfaction, and compliance on the GI and PI scores, and to compare the satisfaction and compliance scores between using the 2 types of toothbrushes.

### LED toothbrush

**Fig. 1:**
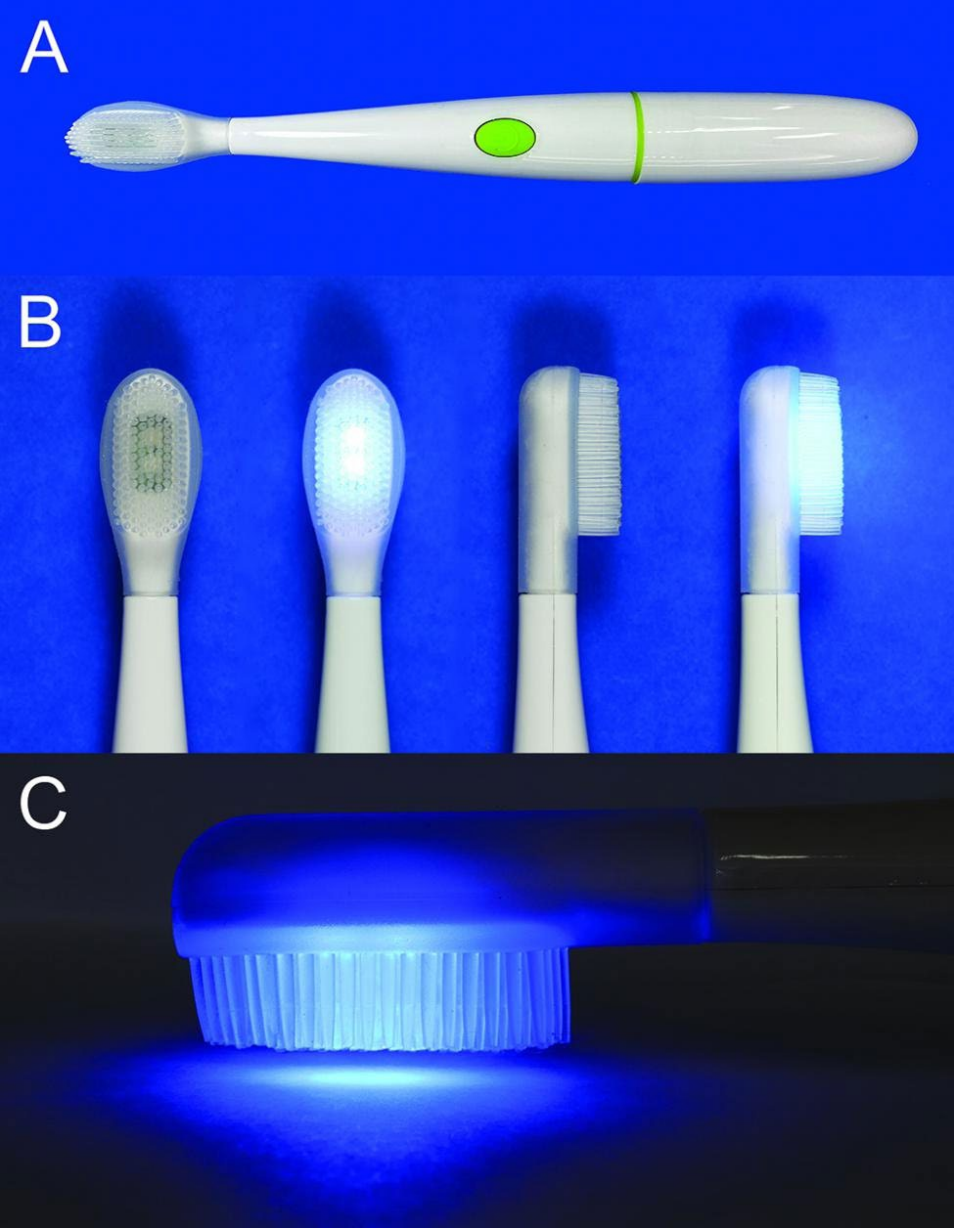
Photographs of a blue light-emitting toothbrush: (**A**) general view, (**B**) toothbrush heads in off and on modes, (**C**) exposure of LED light with 2-mm distance from the surface

The LED toothbrushes (WHITENGO™, UK) used in our study were certified by European Conformity (CE marking) (Fig. 1A, B). The specifications were a 460–480 nm wavelength, 16,000 acoustic pulsations per minute, 840 mW of power, and 1,000–3,000 millicandela of light intensity. The 9-mm long bristles were made from silicone. Five LED toothbrushes were randomly selected for radiance and irradiance measurements with a spectroradiometer (CS-2000, Konica Minolta Inc., Tokyo, Japan) and light intensity meter (MT-4617LED, Pro’sKit Industries Co. LTD, New Taipei, Taiwan), respectively. The radiance was 0.0176 ± 0.0011 W/Sr.m^2^ and the power density was 9.6 ± 1.4 mW/cm^2^.

### Clinical evaluation

#### Trial design, subjects, eligibility criteria, and settings

**Fig. 2:**
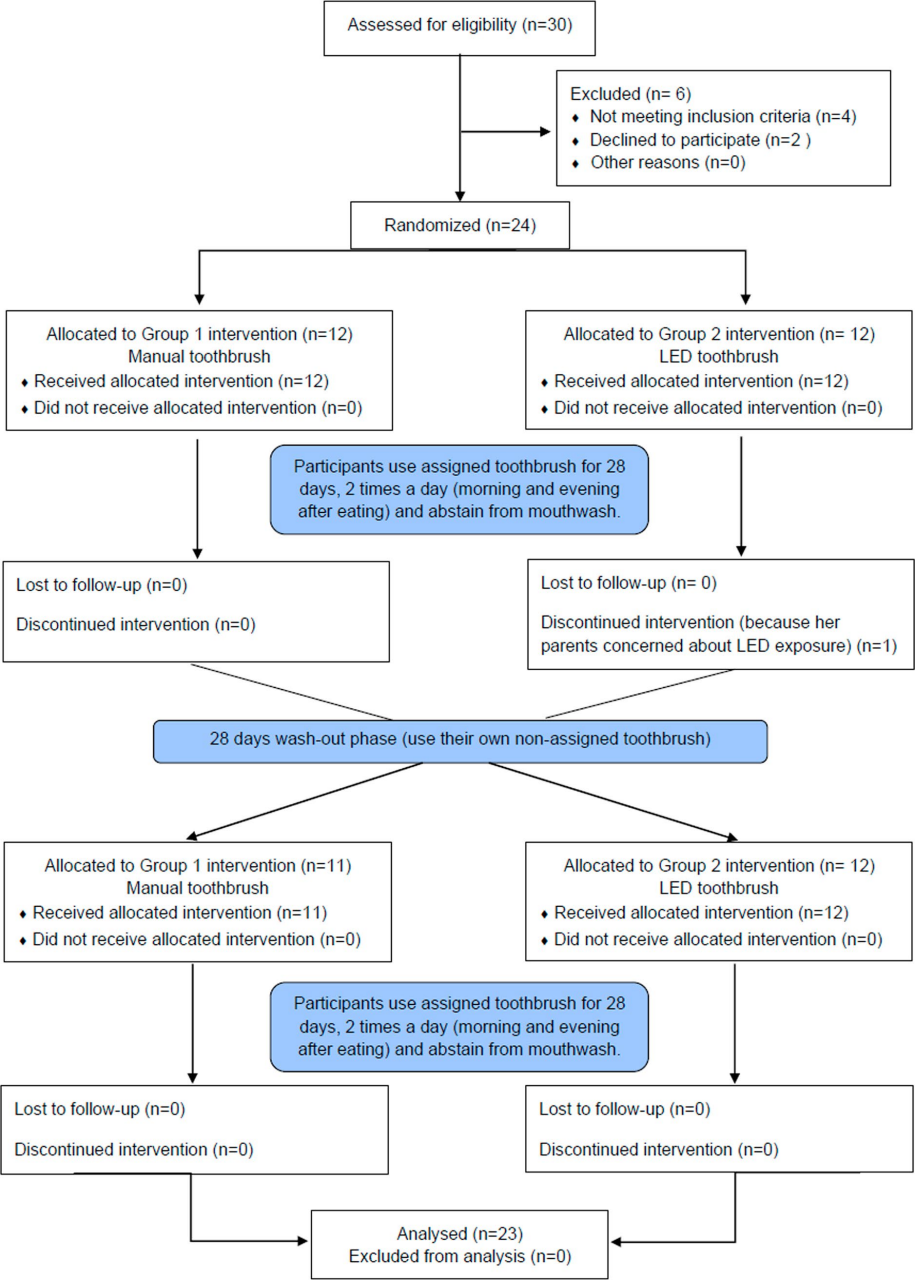
The CONSORT flow diagram: a crossover randomized controlled trial

The null hypothesis is that there is no difference between the blue-light LED toothbrush and manual toothbrush in reducing dental plaque and gingival inflammation in orthodontic patients with fixed appliances. To test this hypothesis, a randomized controlled trial (RCT) with a single-blind two-treatment crossover design was performed. The Consolidated Standards of Reporting Trials (CONSORT) checklist, a guideline for conducting and reporting this trial, is presented in Fig. 2. The study design was approved by the Human Research Ethics Committee of the Faculty of Dentistry, Chulalongkorn University, Thailand (HREC-DCU 2020 − 116), and registered at the Thai Clinical Trials Registry (TCTR; TCTR20210510004) on 10/05/2021. The sample size was calculated from the GI data from a previous study on LED toothbrushes [25] with an alpha of 0.05 and 0.8 power of the test. The calculation indicated that 18 patients were required, which was adjusted to 24 to improve the validity of the study and to compensate for a possible dropout rate of 30% during the follow-up period. The following formula was used to estimate the sample size:

$$n = {({Z_{1 - }}_{\alpha /2} + {Z_{1 - }}_\beta )^2}({\sigma _1}^2 + {\sigma _2}^2)/{\left( {{\mu _1} - {\mu _2}} \right)^2}$$

$$\begin{array}{l}\alpha = 0.05,\beta = 0.2\left( {power = 80\% } \right),dropout = 30\% ,\\{\mu _1} - {\mu _2} = - 0.26,{\sigma _1}{ = _{}}0.30,{\sigma _2} = 0.25\end{array}$$

Twenty-four patients being treated with fixed orthodontic appliances at the post-graduate orthodontic clinic at the Faculty of Dentistry, Chulalongkorn University from October 2021 to June 2022 were recruited. Written informed consent was obtained from the patients before the experimental period. Patients with at least 20 fully erupted permanent teeth, excluding third molars, were being treated with fully bonded fixed appliances, from at least one first molar to the other first molar in the same arch, for more than 1 month, and their remaining irregularity index was less than 1 mm [28] were included in the study. The patients were excluded if they had a systemic disease or other diseases that compromised their hand control or were taking medications that increased or reduced their inflammatory status, such as corticosteroids and nonsteroidal anti-inflammatory drugs. Patients using supplementary plaque control, such as antiseptic mouthwashes or antibiotics within one month before the study, smokers, and patients with periodontitis were also excluded.

#### Randomization and allocation concealment

The 24 subjects were randomly allocated into two groups by block-of-4 randomization. The computer-generated randomization list was created using Microsoft Excel (version 2210, Microsoft Corp., WA, USA). The allocation sequence was concealed in a sealed opaque envelope and kept with a research secretary until the participants finished the second assigned intervention. The allocation was blinded to the outcome assessor (C.M.) as the toothbrushes were given to the participants in a separate room by the secretary. At the end of the trial, the allocation was unveiled by the data analyst (P.C.) who was not involved in the clinical examination.

#### Clinical interventions

In group 1, the patients were assigned to use the manual orthodontic toothbrush (Systema OD, Lion Company, Tokyo, Japan) for 28 d, followed by using their own non-assigned toothbrush for 28 d (wash-out period), and then used the LED toothbrush for 28 d. In group 2, the patients used the LED toothbrush during the first period (28 d), wash-out for 28 d, and then used the manual toothbrush during the second period (28 d). The patients were instructed by one dentist for 5 min on how to brush with the Bass technique before starting the interventions [29]. To clean the area around the brackets, the patients were instructed to place the bristles on the top and the bottom of the brackets and brush them with horizontal strokes. Both groups used the same toothpaste (Sensodyne repair and protect, GSK™, Thailand). The patients brushed with their assigned toothbrush twice a day, in the morning and in the evening after eating, for 28 d. During the intervention periods, any perceived side effects could be directly reported to the examiner. The patients were informed that they could stop their participation at any time.

#### Primary and secondary outcomes

In the present study, the GI score was considered as the primary outcome, and the PI score, patients’ compliance score, and satisfaction score of the toothbrushes were examined as the secondary outcomes. The GI and PI scores, following Loe-Silness’s criteria [30, 31] which range from 0 to 3, were collected at baseline and after 28 d of each intervention period. Before starting the experiment, two trained examiners were calibrated for evaluating the GI and PI by assessing both indices on 3 patients with fixed orthodontic appliances.

**Fig. 3:**
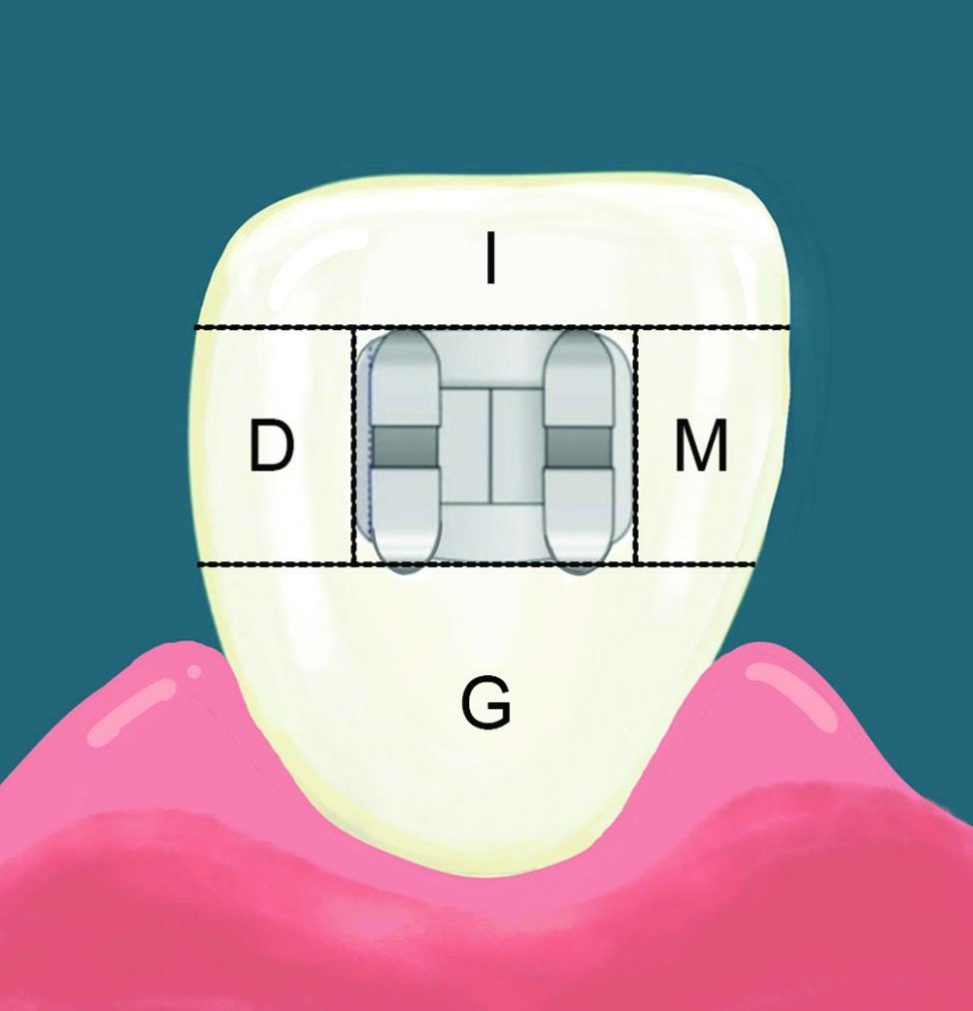
Schematic diagram of the tooth surface areas used for the plaque index on the bracket side: (I) incisal, (G) gingival, (M) mesial, and (D) distal(29)

The GI and PI scores were collected on Ramfjord’ six representative teeth [32], i.e., the maxillary right first molar, maxillary left central incisor, maxillary left first premolar, mandibular left first molar, mandibular right central incisor, and mandibular right first premolar. In case of a missing first premolar or first molar, the second premolar or second molar, respectively, was used as a substitute. The indices were evaluated on the bracket and non-bracket sides. For the PI on the bracket sides, 4 zones around the brackets, mesial (M), distal (D), gingival (G), and incisal (I) were measured (Fig. 3), as previously described for evaluating dental plaque in orthodontic patients [33]. In the crossover design, the GI and PI scores were evaluated 4 times for each participant, i.e., (1) before the first intervention (baseline-1); (2) after the first intervention (28 days-1); (3) after the washout period, which is before the second intervention (baseline-2); and (4) after the second intervention (28 days-2). The baseline and 28-days values of each intervention were combined from the two groups of participants for statistical analysis.

The intra-examiner and inter-examiner reliability were evaluated by the intraclass correlation (ICC). The evaluation was done twice within a 4-week interval. For the PI score, the ICC values were 0.948 for the intra-examiner, and 0.799 for the inter-examination agreement. For the GI score, the ICC values were 0.972 for the intra-examiner, and 0.785 for the inter-examination agreement.

The patients’ compliance and satisfaction with the toothbrushes were evaluated via a questionnaire. The patients’ compliance scores (score 0–4) were rated as the frequency of toothbrush use; 0 – never, 1 – rarely, 2 -sometimes, 3 – often, and 4 – always. The patients’ satisfaction with each type of toothbrush (score 0–3) were scored as 0 – not satisfied, 1 – slightly satisfied, 2 – moderately satisfied, and 3 – very satisfied.

#### Blinding

Double blinding was not possible in the present study because the participants could not be blinded to the interventions. However, the outcome assessor was blinded to the interventions to which the patients were assigned.

### In vitro evaluation

**Fig. 4:**
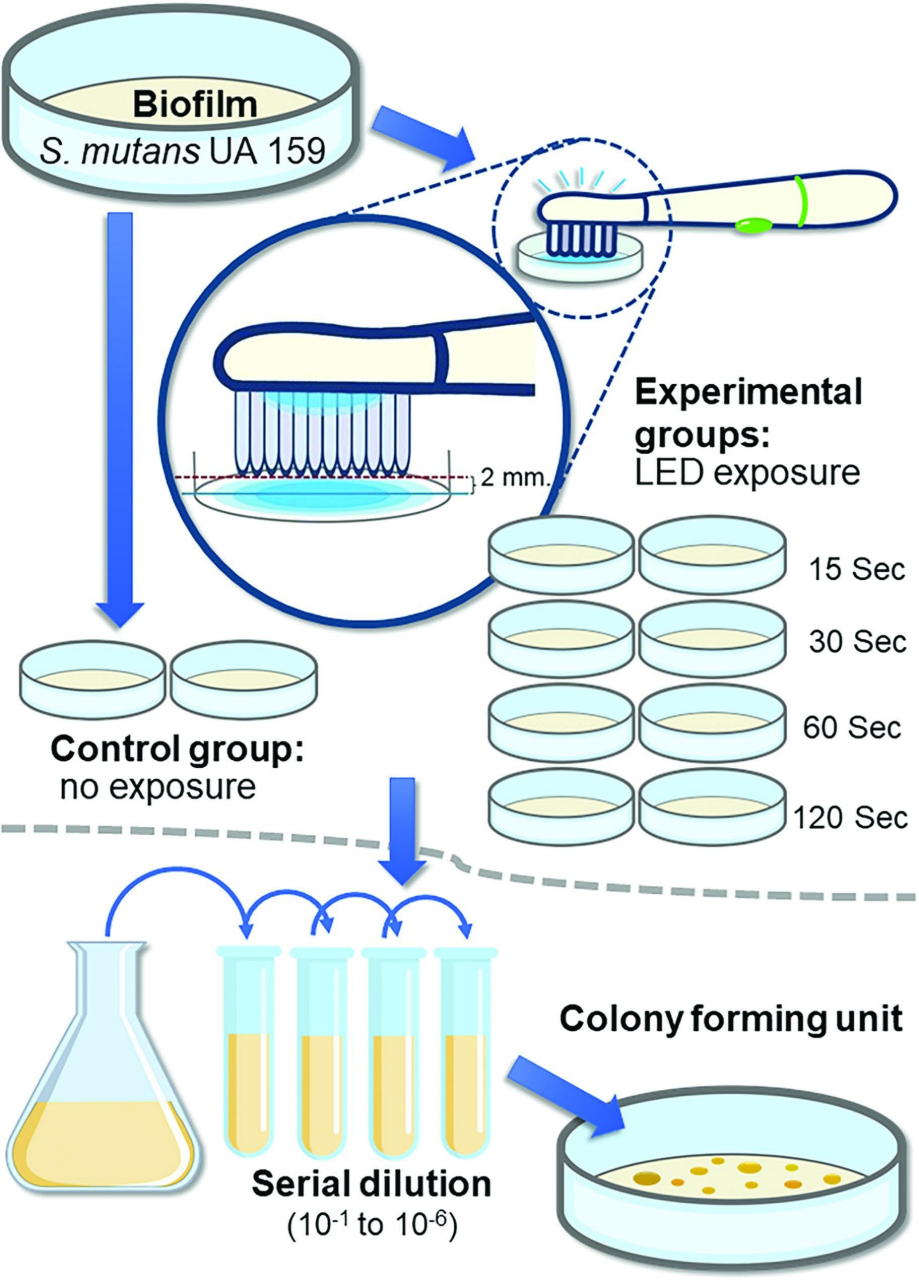
In vitro experimental flow. The experiments were repeated 3 times (total n = 6/group)

Our study also evaluated the effect of LED toothbrushes on *S. mutans* biofilm in vitro. The experimental flow and study design are shown in Fig. 4.

#### Bacterial strains and growth conditions

*S. mutans* UA159 from bacterial glycerol stocks were inoculated in Brain-Heart Infusion (BHI) agar, and incubated at 37 °C in 5% CO_2_ for 24 h. An isolated colony was regrown overnight in BHI broth with sustained shaking at 240 rpm. Next, its optical density at 600 nm (OD 600 nm) was determined and adjusted until reaching a value of 0.1. The culture was incubated at 37 °C in 5% CO_2_ for 3 h to reach the logarithmic phase of growth (OD 600 nm ≈ 0.4–0.6) that was used for biofilm formation.

#### Biofilm formation

The bacterial cells at the log phase were harvested by centrifugation (12,000 xg, 4 °C, 15 min). The cells were resuspended in BHI broth with 1% sucrose. A 3 mL suspension (3 × 10^8^ bacterial cells) was placed in 35-mm dishes and incubated at 37 °C in 5% CO_2_ for 36 h.

#### Exposure of the biofilm to the LED toothbrush

After incubation, the supernatant above the biofilm was removed. The biofilm samples were divided into 5 groups based on the LED exposure time, with 2 biofilm plates in each group. The control group did not receive LED exposure. The other four experimental groups were exposed to the LED toothbrush for 15, 30, 60, or 120 s. To avoid the effect of the acoustic pulsations, the LED toothbrush was switched on and held 2 mm above the biofilm (Figs. 1C and 4).

The biofilm was scraped off and put in 100 µl sterile PBS. The bacteria suspensions were sonicated and serially diluted (10^− 1^–10^− 6^). Next, 100 µl of each concentration was dropped onto BHI agar in duplicate and incubated for 36 h at 37 °C in 5% CO_2_. The highest concentration from which 30–300 individual colonies could be counted was used to calculate the number of bacteria. The percentage of bacterial survival was calculated relative to the control. The experiment was repeated 3 times (total n = 6/group) (Fig. 4).

### Statistical analysis

The data was collected and analyzed using statistical software (SPSS, version 22.0, IBM, IL, USA). For the in vitro data, the Kruskal-Wallis test was used to test the difference in the percentage of bacteria viability among the groups. Post hoc comparisons were performed using the Mann-Whitney U test with Bonferroni correction for multiple pairwise comparisons. For the clinical trial data, the Shapiro-Wilk test was used to determine the normality of the data. After 28 d, the GI and PI values of the 2 interventions were compared using analysis of covariance (ANCOVA), including their baseline scores as a covariate. The influence of potential confounders on the GI and PI was evaluated by linear regression. The satisfaction and compliance levels between the 2 types of toothbrushes were compared by the Wilcoxon Signed Rank test. The correlations between the order of the assigned toothbrushes, patients’ age, compliance scores, and satisfaction scores were evaluated using Spearman’s correlation coefficient. Significant differences were defined at *P* < 0.05.

## Results

### Clinical evaluation

The trial ended in September 2022, without any adverse effect observed in the participants. Of the 24 participants, one dropped out during the first period because the patient had concerns about the side effects of LED light. Thus, 23 subjects (5 males and 18 females) with an age range of 16–31 years old (mean 21.2 ± SD 5.1) were analyzed in this study. The baseline demographic and characteristics of the participants are shown in Table 1.

**Table 1:** Baseline demographic, characteristics, and clinical data of trial participants

Characteristics			Bracket sides	Non-bracket sides
**Sex**, n (%)	Female	18 (78.3)		
	Male	5 (21.7)		
**Age**; Mean (SD)		21.2 (5.1)		
**Baseline** **Gingival Index**; n (%)	0		0 (0)	0 (0)
	1		10 (43.5)	11 (47.8)
	2		13 (56.5)	12 (52.2)
	3		0 (0)	0 (0)
**Baseline** **Plaque Index**; n (%)	0		0 (0)	0 (0)
	1		2 (8.7)	3 (13.1)
	2		19 (82.6)	19 (82.6)
	3		2 (8.7)	1 (4.3)

#### Gingival index and plaque index scores

The means and standard errors of the GI and PI values at baseline and after 28 d of the interventions are presented in Table 2. The reductions in the GI score after 28 d were slightly greater in the LED group compared with the manual toothbrush group. The mean differences on the bracket and non-bracket sides were − 0.008 and − 0.009, respectively. However, no significant difference was found between using the 2 types of toothbrushes (*P* = 0.930 on the bracket side, *P* = 0.913 on the non-bracket side).

**Table 2:** Gingival Index (GI) and Plaque Index (PI) on the bracket and non-bracket sides before and after brushing using each toothbrush and their differences

Side	Site	Visit	Mean (SE)		Mean Diff ^a,b^	95% CI ^a^	% diff ^c^		*P*-value ^a^
			Manual	LED			Man	LED	
Gingival Index									
Bracket	All surface	Baseline	1.40 (0.08)	1.49 (0.07)					
		28 days	1.37 (0.07)	1.41 (0.07)	− 0.008	− 0.195, 0.178	-0.57	-0.54	0.930
		Post-Pre (Δ)	-0.03 (0.08)	-0.09 (0.07)					
Non-bracket	All surface	Baseline	1.42 (0.08)	1.52 (0.08)					
		28 days	1.40 (0.07)	1.44 (0.07)	− 0.009	− 0.174, 0.156	-0.63	-0.59	0.913
		Post-Pre (Δ)	-0.02 (0.06)	-0.08 (0.07)					
**Plaque Index**									
Bracket	Proximal	Baseline	1.77 (0.09)	1.64 (0.10)					
		28 days	1.35 (0.11)	1.56 (0.10)	0.286	0.027, 0.545	16.16	17.44	0.031*
		Post-Pre (Δ)	-0.42 (0.12)	-0.08 (0.07)					
	Gingival	Baseline	1.80 (0.09)	1.74 (0.11)					
		28 days	1.46 (0.10)	1.50 (0.10)	0.072	− 0.170, 0.314	4.00	4.14	0.553
		Post-Pre (Δ)	-0.34 (0.12)	-0.24 (0.07)					
	Incisal	Baseline	0.97 (0.09)	0.94 (0.10)					
		28 days	0.73 (0.08)	0.82 (0.09)	0.109	− 0.109, 0.327	11.24	11.60	0.321
		Post-Pre (Δ)	-0.24 (0.08)	-0.12 (0.10)					
	All site average	Baseline	1.58 (0.08)	1.49 (0.10)					
		28 days	1.23 (0.09)	1.36 (0.09)	0.186	− 0.038, 0.409	11.77	12.48	0.101
		Post-Pre (Δ)	-0.36 (0.10)	-0.13 (0.07)					
Non-bracket	All surface	Baseline	1.86 (0.07)	1.96 (0.07)					
		28 days	1.74 (0.09)	1.76 (0.09)	− 0.056	− 0.256, 0.144	-3.01	-2.86	0.576
		Post-Pre (Δ)	-0.12 (0.08)	-0.19 (0.06)					

^a^From ANCOVA analysis, ^b^Mean Diff = LED – Manual (based on estimated marginal means), ^c^% diff = percentage calculated as (mean difference/mean of baseline) x 100, 95% CI = 95% confidence interval, ^*^Significant difference (*P* < 0.05)

Similarly, the PI scores after 28 d were not significantly different between the groups on the bracket (*P* = 0.101) or non-bracket sides (*P* = 0.576). When the different tooth surface areas of the bracket side were analyzed separately, the manual toothbrushes significantly reduced the PI score in the proximal area (23.7%), compared with that of the LED toothbrush (4.8%, *P* = 0.031). In contrast, the post-treatment PI scores were not significantly different between the groups in the gingival and incisal areas (*P* = 0.553 and 0.321, respectively).

The univariate linear regression analysis determining the effect of the confounders on the GI and PI scores revealed that the participants’ age was identified as a significant predictor for the GI score on the non-bracket side (*P* = 0.032). However, the order of the assigned toothbrushes, satisfaction scores, and compliance scores did not influence the GI and PI values. Moreover, the change in the PI had no effect on the change in the GI. The multivariate model confirmed that there was no association among the potential confounders that influenced the treatment outcomes (Table 3).

**Table 3:** Linear regression model analysis of the influence of potential confounders on the gingival index (GI) and plaque index (PI)

Variables		Univariate model				Multivariate model			
		R^2^	β (SE)	*Std* *Co*	*P-v*alue	R^2^	β (SE)	*Std* *Co*	*P-v*alue
**ΔGI** **Bracket side**	Age	0.007	0.006 (0.011)	0.086	0.571	0.094	0.009 (0.011)	0.128	0.408
	Order of toothbrushes	0.001	− 0.011 (0.046)	− 0.036	0.811		− 0.029 (0.052)	− 0.093	0.582
	Satisfaction score	0.003	0.036 (0.098)	0.055	0.715		0.019 (0.105)	0.029	0.857
	Compliance score	0.010	0.063 (0.094)	0.101	0.505		0.024 (0.098)	0.039	0.805
	**Δ**Plaque index	0.064	0.212 (0.122)	0.254	0.089		0.243 (0.134)	0.291	0.077
**ΔGI** **Non-Bracket side**	Age	0.100	0.021 (0.009)	0.316	0.032*	0.124	0.018 (0.010)	0.280	0.074
	Order of toothbrushes	0.005	− 0.021 (0.43)	− 0.072	0.636		− 0.012 (0.045)	− 0.043	0.784
	Satisfaction score	0.016	0.077 (0.090)	0.128	0.398		0.095 (0.094)	0.157	0.318
	Compliance score	0.002	0.026 (0.087)	0.044	0.771		0.026 (0.089)	0.045	0.773
	**Δ**Plaque index	0.044	− 0.207 (0.146)	0.0209	0.163		− 0.176 (0.158)	0.178	0.269
**ΔPI** **Bracket side**	Age	0.021	− 0.012 (0.013)	− 0.146	0.334	0.117	− 0.013 (0.012)	− 0.150	0.316
	Order of toothbrushes	0.062	0.092 (0.054)	0.250	0.094		0.111 (0.058)	0.300	0.062
	Satisfaction score	0.000	0.005 (0.118)	0.006	0.967		0.051 (0.122)	0.066	0.677
	Compliance score	0.014	0.089 (0.112)	0.119	0.431		0.127 (0.112)	0.170	0.265
**ΔPI** **Non-Bracket side**	Age	0.068	− 0.017 (0.010)	− 0.260	0.081	0.162	− 0.018 (0.010)	− 0.270	0.067
	Order of toothbrushes	0.027	− 0.048 (0.043)	-165	0.274		− 0.090 (0.044)	− 0.104	0.498
	Satisfaction score	0.022	0.090 (0.091)	0.147	0.330		0.034 (0.093)	0.055	0.718
	Compliance score	0.069	0.153 (0.085)	0.262	0.079		0.148 (0.086)	0.252	0.092

SE – standard error, Std Co - standardized coefficients, ^*^Significant difference (*P* < 0.05)

#### Patients’ compliance and satisfaction

The patients’ compliance and satisfaction scores when using the manual toothbrushes were slightly higher than those when using the LED toothbrushes, however, the differences were not significant. The satisfaction scores when using the manual and LED toothbrushes ranged from 2 to 3 (mean 2.6 ± SD 0.5), and 1–3 (mean 2.3 ± SD 0.5), respectively. The questionnaires revealed that 11 patients (47.82%) were more satisfied with the manual toothbrushes, 8 patients (34.78%) were indifferent to the toothbrush type, and 4 patients (17.39%) preferred the LED toothbrushes to the manual toothbrushes. Moreover, the compliance scores when using the manual toothbrush ranged from 3 to 4 (mean 3.8 ± SD 0.4), while the scores when using the LED toothbrush ranged from 2 to 4 (Mean 3.5 ± SD 0.7).

#### Correlations between the patients’ age, order of the assigned toothbrushes, satisfaction, and compliance

A positive correlation was found between the patients’ age and the compliance scores for the manual toothbrush (*P* = 0.028), however, there was no correlation with the LED toothbrush. In addition, patient compliance when using the manual toothbrush was correlated with those using the LED toothbrush. The order of the toothbrushes did not influence the patients’ compliance or satisfaction (Table 4).

**Table 4:** The correlation coefficients between the patients’ age, order of the assigned toothbrushes, satisfaction scores, and compliance scores

Variables		Age	Order of toothbrushes	Satisfaction Manual	Satisfaction LED	Compliance Manual	Compliance LED
**Age**	Corr Coef	1.000	− 0.172	− 0.020	− 0.164	0.457^*^	− 0.100
	Sig.	.	0.433	0.927	0.455	0.028	0.649
**Order of toothbrushes**	Corr Coef	− 0.172	1.000	− 0.233	− 0.346	− 0.083	0.114
	Sig.	0.433	.	0.285	0.106	0.708	0.605
**Satisfaction** **Manual**	Corr Coef	− 0.020	− 0.233	1.000	− 0.274	0.225	− 0.140
	Sig.	0.927	0.285	.	0.206	0.301	0.524
**Satisfaction** **LED**	Corr Coef	− 0.164	− 0.346	− 0.274	1.000	− 0.133	− 0.049
	Sig.	0.455	0.106	0.206	.	0.544	0.823
**Compliance Manual**	Corr Coef	0.457^*^	− 0.083	0.225	− 0.133	1.000	0.441^*^
	Sig.	0.028	0.708	0.301	0.544	.	0.035
**Compliance LED**	Corr Coef	− 0.100	0.114	− 0.140	− 0.049	0.441^*^	1.000
	Sig.	0.649	0.605	0.524	0.823	0.035	.

Corr Coef = Correlation coefficient, Sig. (2-tailed), ^*^Significant difference (*P* < 0.05)

### Harms

No harm or adverse effect were recorded in our study.

### In vitro evaluation

#### Effect of LED toothbrush on mature Streptococcus mutans biofilm

**Fig. 5:**
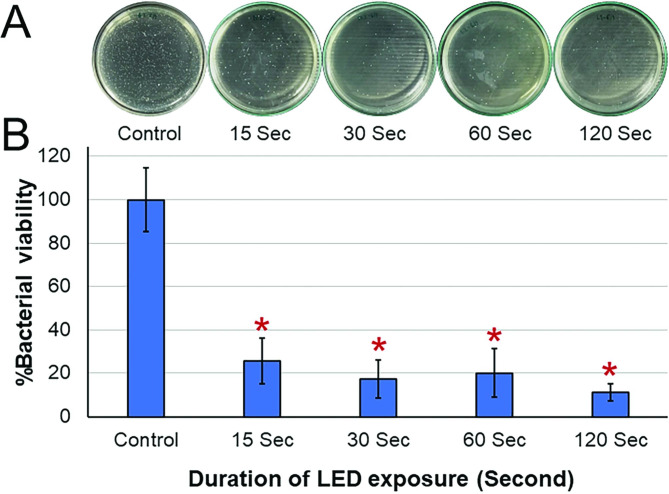
Effect of the LED toothbrush on *Streptococcus mutans* biofilm: (**A**) Colony forming Units, (**B**) The comparison of percentage of bacterial viability. The group with 15-, 30-, 60-, and 120-sec exposure had a significantly lower percentage of bacterial viability than the negative control group (no LED) (*P* < 0.05). Asterisks indicate a significant difference compared with no LED group. No significant difference was detected between the exposure time points

The percentage of bacterial viability was significantly different among the groups (*P* = 0.006). Pairwise comparisons indicated that the 15-, 30-, 60-, and 120-sec exposure groups had a significantly lower percentage of bacteria viability compared with the control group (no LED exposure). The 120-sec group had the lowest percentage of bacteria viability. However, there was no significant difference between the experimental groups (Fig. 5A, B).

## Discussion

Our clinical study was performed as a single-blind crossover RCT with a 28-d brushing period to compare the effectiveness of an LED toothbrush with a manual toothbrush in reducing dental plaque and gingival inflammation. A randomized design was used to minimize bias error. The crossover trial allowed us to compare the outcomes from using 2 different types of toothbrushes within the same participants. Because the crossover RCT eliminates the variation among participants, with the same sample size, the results from the 2 interventions can be determined with greater precision, compared with a parallel group trial in which each participant is exposed to only one intervention [34].

We recruited patients who were undergoing fixed orthodontic appliances for more than 1 month because this allowed time for the patients to adapt their brushing skills with the fixed orthodontic appliance, thus, the outcomes were not influenced by the time-dependent acquisition of brushing skills. In addition, to exclude the variation in dental crowding from confounding the results, only participants who had an irregularity index less than 1 mm were recruited. We excluded participants with a higher irregularity index because the crowding may be considerably resolved by the treatment during the period between the first and second interventions, which was 56 days or 2 orthodontic treatment visits. This might affect the outcomes of the interventions and could lead to a sequence effect in the crossover design. Furthermore, dental crowding might limit the LED light exposure to the dental plaque in the crowded areas. The patients performed their assigned brushing methods at home to simulate the normal condition. At baseline, the GI and PI scores were not significantly different between group 1 and 2. Overall, the LED toothbrush use did not significantly reduce the GI and PI scores compared with the manual toothbrush. However, the GI and PI scores decreased in both toothbrush groups. This might be explained by the Hawthorne effect where patients change their behavior because they knew that they would be observed [35]. The reinforced instruction of the Bass technique might also have helped.

Previous studies on the effectiveness of an LED toothbrush were conducted in non-orthodontic patients. A study using a blue LED toothbrush (405–420 nm wavelength and 2 mW/cm^2^ power density) reported significantly reduced dental plaque, gingival bleeding, and inflammation compared with the manual toothbrush after using them for one month [16]. Another study found that the GI and bleeding on marginal probing were significantly lower in the LED electric toothbrush group compared with the non-LED group after 6 weeks [25]. This study used the advanced version Electric 3-color LED toothbrush that has 2 blue lights, 1 red light, and 1 white light with a power density of 16.5–18.5 mW/cm^2^. In contrast, another study using a blue LED light toothbrush with a 450 nm wavelength and 13.5 mW/cm^2^ power density indicated that it did not have a significant effect in reducing dental plaque and gingival inflammation compared with the toothbrush without the LED blue light [36]. The contradictory results from these studies might be due to the variations in the light colors, wavelengths, and power density of the LED toothbrushes, which influenced the bactericidal effect of an aPDT. In our study, we used LED toothbrushes that projected only 460–480 nm blue light with 9.6 mW/cm^2^ power density and evaluated them in fixed orthodontic patients. Our results suggest that blue light only might not be sufficient to deliver the bactericidal effect on light-inaccessible areas, such as beneath the bracket wings and archwires. In addition, the PI baseline score in our study was lower than the 3-colored LED light study, so it might be more difficult to observe an improvement.

The results of our study demonstrated that the LED toothbrush effectively reduced the PI score on the non-bracket side. The reduction in dental plaque was 1.6-fold higher in the LED group, compared with the manual group. In contrast, the opposite outcomes were found on the bracket side. The metal brackets and archwires might limit the LED light exposure to the dental plaque. These results imply that the LED toothbrush successfully reduced dental plaque by inhibiting bacterial viability in the light-accessible area. In addition, the clinical results in the present study revealed that the LED light was less effective compared with the in vitro results. This might also be because when brushing, the participants used toothpaste that might reduce the LED rays so that the oral biofilm was not maximally exposed to the LED light. Perhaps an alternative toothbrush design, where the light is placed outside of the bristle zone might be better because the LED light would not be blocked by the bristles and toothpaste when brushing.

Our results revealed that the manual toothbrush performed better than the LED toothbrush in reducing dental plaque in the proximal areas on the bracket side. In accordance with the patients’ comments in our questionnaires, most of the patients who preferred the manual toothbrush complained that the LED toothbrush was larger than their usual toothbrush. Thus, the LED toothbrushes were more difficult to hold and brush in restricted spaces like the proximal areas. Our results also revealed that patients who were compliant were equally compliant with using the manual toothbrush and the LED toothbrush. Additionally, older patients were significantly more compliant with the manual toothbrush than younger patients. In this study, the participants’ age was the only confounder that had the influence over a treatment outcome, which was the change in GI score on the non-bracket side. According to previous studies, the inflammatory response in gingival tissue to dental plaque is higher in older patients [37, 38].

When using aPDT, the mechanism of the bactericidal effect of light is that the bacteria contains a photosensitizer, an agent that absorbs the light [39]. When the photosensitizer is activated by light with its preferred wavelength, electrons are transferred to produce free radical ions that react with oxygen and result in cytotoxic or reactive oxygen species [40]. At the oral microbiota level, periodontal pathogens, such as *Prevotella nigrescens* and *Prevotella intermedia*, contain endogenous porphyrins [41], which are photosensitizers. Moreover, *S. mutans*, a dominant member of the cariogenic flora, has been investigated using many exogenous photosensitizers, such as erythrosine, toluidine blue, and malachite green [42–44]. However, the specific endogenous photosensitizer has not been determined.

The activity of blue light against *S. mutans* has been demonstrated in previous in vitro studies [15, 22, 45]. Blue light was reported to reduce *S. mutans* biofilm reformation [22]. In our study, we evaluated the bactericidal effect of LED toothbrush on mature *S. mutans* biofilm in vitro by varying the time exposure from 0 to 120 s, without an exogenous photosensitizer. Although the light passed through the 9-mm long silicon bristles, it still had a bactericidal effect on *S. mutans* biofilm after a 15-sec exposure in vitro.

The exposure time is one of the most important factors for the bactericidal effect of an LED toothbrush. Previous in vitro studies [15, 22, 45] set the exposure duration from 1 to 10 min. However, people usually brush their teeth for 2–3 min, and the toothbrush does not stay at one position for minutes. Therefore, our study varied the duration of blue light exposure to determine the shortest time that the blue light could significantly decrease the viability of *S. mutans.* Our data indicated that a significant reduction in bacterial viability occurred after the biofilm had been exposed to blue LED light for at least 15 s. The viability was reduced by 75% after a 15-sec exposure. Increased exposure time resulted in an increased percentage of bactericidal effect. However, the LED toothbrush in our study was used on biofilm formed in 35-mm dishes, which are approximately equal to 2–3 teeth and the size of the LED toothbrush. Thus, if an LED toothbrush was used for at least 15 s on 2–3 teeth, it may reduce the bacterial viability on the tooth surface. However, the full-mouth brushing time of 2–3 min that is generally recommended by dental associations [46] might not be enough [47]. Based on our in vitro results, we suggest that a longer brushing time might increase the bactericidal effect of the LED toothbrush.

Overall, our study adds clinical evidence concerning the effectiveness of the LED toothbrushes, especially for fixed orthodontic patients. However, we tested only the blue-light LED toothbrush, and our in vitro study was performed only on *S. mutans* biofilm. One of the limitations of this study was its sample size, because of the several lockdown periods due to the COVID-19 pandemic. Moreover, the bristle material of the LED toothbrush was made of silicone instead of nylon like the manual toothbrush. According to the participants’ feedback, silicone bristles were softer and less springy. These differences might reduce the effectiveness of the LED toothbrush, especially when brushing on the bracket side. Furthermore, *S. mutans* does not completely represent the dental plaque, which is a multispecies biofilm composed of a complex bacterial population, saliva constituents, and a polymer matrix. The various species of bacteria might have antibacterial susceptibility to different wavelengths and exposure times [22]. Furthermore, there are few previous studies about the antimicrobial effect of an LED toothbrush. Consequently, there was a lack of data for our research planning and discussion.

Future clinical studies with different light colors from an LED toothbrush, an exogenous photosensitizer, or new LED toothbrush designs would be beneficial for more solid evidence, especially in orthodontic patients with fixed appliances. Moreover, in vitro investigations using other bacterial strains or more complex biofilms should be performed.

## Conclusion

Even though, the blue light from the LED toothbrush significantly reduced the amount of *S. mutans* in biofilm in vitro when the biofilm was exposed to the light for at least 15 s, the LED toothbrush was not more effective than the manual toothbrush in reducing dental plaque and gingival inflammation, especially in the proximal areas. Therefore, our results suggest that the blue-light LED toothbrush is not a better choice to use in orthodontic patients with fixed appliances.

## Electronic supplementary material

Below is the link to the electronic supplementary material.

Supplementary Material 1

## Data Availability

The datasets used and/or analyzed during the current study are included in this published article and the supplementary information files.

## Abbreviations

LED: Light emitting diode
*S. mutans*: *Streptococcus mutans*
aPDT: Antimicrobial photodynamic therapy
PI: Plaque index
GI: Gingival index
